# Synonymous Codon Usage Analysis of Thirty Two Mycobacteriophage Genomes

**DOI:** 10.1155/2009/316936

**Published:** 2010-02-01

**Authors:** Sameer Hassan, Vasantha Mahalingam, Vanaja Kumar

**Affiliations:** Tuberculosis Research Centre Indian Council of Medical Research, Chennai 600 031, India

## Abstract

Synonymous codon usage of protein coding genes of thirty two completely sequenced mycobacteriophage genomes was studied using multivariate statistical analysis. One of the major factors influencing codon usage is identified to be compositional bias. Codons ending with either C or G are preferred in highly expressed genes among which C ending codons are highly preferred over G ending codons. A strong negative correlation between effective number of codons (Nc) and GC3s content was also observed, showing that the codon usage was effected by gene nucleotide composition. Translational selection is also identified to play a role in shaping the codon usage operative at the level of translational accuracy. High level of heterogeneity is seen among and between the genomes. Length of genes is also identified to influence the codon usage in 11 out of 32 phage genomes. Mycobacteriophage Cooper is identified to be the highly biased genome with better translation efficiency comparing well with the host specific tRNA genes.

## 1. Introduction

The genetic code uses 64 codons to represent the 20 standard amino acids and the translation termination signal. Each codon is recognized by a subset of a cell's transfer ribonucleotide acid molecules (tRNAs), and with the exception of a few codons that have been reassigned in some lineages [1, 2] the genetic code is remarkably conserved, although it is still in a state of evolution [3].

Codons can be grouped into 20 disjoint families and each family in the universal genetic code contains between 1 and 6 codons. The usage of alternate synonymous codons in an organism is understood to be nonrandom. Grantham et al. [4, 5] proposed that each genome has a particular codon usage signature that reflects particular evolutionary forces acting within that genome. The synonymous codons and amino acids are not used at equal frequencies both within and between organisms [5–7]; the patterns of codon usage vary considerably among organisms, and also among genes from the same genome [8]. 

Several factors such as directional mutational bias [9–11], translational selection [11–15], secondary structure of proteins [16–21], replicational and transcriptional selection [22, 23], and environmental factors [24, 25] have been reported to influence the codon usage in various organisms. In contrast, amino acid usage has been shown to be influenced by factors such as hydrophobicity, aromaticity, cysteine residue (Cys) content, and mean molecular weight (MMW) [24, 26–30]. Compositional constraints and translational selection are thought to be the two main factors for the codon usage variation among the genes in and across genomes. Compositional bias shapes the codon usage variation among the genes in the extremely AT or GC rich unicellular organisms [31–33]. Analysis of codon usage pattern can provide a basis for understanding the relevant mechanism for biased usage of synonymous codons [34] and has both practical and theoretical importance in understanding the basics of molecular biology [7, 35–39]. 

Bacteriophages generally use the translational machinery of their hosts to synthesize both their structural and regulatory proteins. This indicates that the amount of codon usage in the protein coding genes in the phages and their bacterial hosts should be similar. 

Mycobacteriophages have the potential to be used in diagnosis of tuberculosis and as molecular tools to study mycobacteria. Understanding the codon usage pattern of these phages should guide in the selection of appropriate ones for such purposes. The present venture is to study and understand codon usage patterns of all the mycobacteriophages so far sequenced.

Codon usage analysis was previously done for fourteen phages by Sau et al. [40]. Eighteen more mycobacteriophage genomes were subsequently sequenced and became available in Genbank. In the present work we have analysed all these 32 phage genomes to study and compare their codon usage pattern. Other codon usage indices that affect the genomes of these phages are also studied.

## 2. Materials and Methods

### 2.1. Sequences

The complete genome sequences of 32 mycobacteriophages were downloaded from GenBank. Genes having more than 100 codons with proper start and stop codons and without any intermediate stop codon were selected for the current study.

### 2.2. Analysis

Numbers of codons (Ncs), Relative Synonymous Codon Usage (RSCU), and GC composition at every position of codons were calculated for each gene. The analysis was carried out by GCUA [41], CODONW 1.4.2 (http://codonw.sourceforge.net/).

- Nc, the “effective number of codons” used in a gene measures the bias away from equal usage of codons within synonymous groups [19]. Nc can take values from 20 to 61, when only one codon or all synonyms in equal frequencies were used per amino acid, respectively. Nc appears to be a good measure of general codon usage bias [19, 42]. The sequences in which Nc values are <30 are highly expressed while those with >55 are poorly expressed genes [12, 43].
- Relative Synonymous Codon Usage. Relative synonymous codon usage (RSCU) is defined as the ratio of the observed frequency of codons to the expected frequency if all the synonymous codons for those amino acids are used equally [21]. RSCU is used to observe the synonymous codon usage variation among the genes.
- Base composition. The frequency of A, T, G, C, and GC at first, second, and third positions of synonymously variable sense codons which can potentially vary from 0 to 1 was calculated. The variation of GC3s among genes was characterized by its standard deviation.

### 2.3. Statistical Methods

Correspondence analysis (CA) is used to study the codon usage variation between genes in different organisms in which the data are plotted in a multidimensional space of 59 axes excluding those of Met, Trp, and stop codons [19]. For understanding the codon usage variation of mycobacteriophages chosen for the current study, RSCU values are used for CA in order to minimize the amino acid composition. To investigate the difference between high and low expressed genes, we have compared the codon usage variation between 10% of the genes located at the extreme right of axis 1 and 10% of the genes located at the extreme left of the axis 1 produced by CA using RSCU. To estimate the codon usage variation between these two sets of genes we have performed Chi square tests taking *P* < .01 as significant criterion.

The Pearson correlation coefficient and linear regression were calculated to identify the indices that influence the codon usage variation in mycobacteriophages using SPSS version 10.0. The levels of statistical significance were defined as *P* < .01 or *P* < .05.

## 3. Results and Discussion

### 3.1. Overall Codon Usage Analysis in Mycobacteriophages

The RSCU values of 32 mycobacteriophage genomes show that G- and/or C-ending codons are predominantly used (Table 1), in which 13 are C-ending and 6 are G-ending codons. This was expected, as these phages have a high genomic content. However, from the overall RSCU values, it can be assumed that compositional constraint is the only factor responsible for shaping the codon usage variation among the genes in these genomes. Although the overall RSCU values could unveil the codon usage pattern for the genomes, it may hide the codon usage variation among different genes in a genome.

### 3.2. Codon Usage Variation in 32 Mycobacteriophages

The codon usage bias in the coding regions of 32 completely sequenced mycobacteriophages of varying G + C content has been investigated. The average values of the effective numbers of codon (Nc) in different mycobacteriophages varied from 31.44 to 47.96 in mycobacteriophage Cooper and mycobacteriophage Barnyard, respectively. Nucleotide usage pattern in third codon position of all the mycobacteriophages showed high codon usage variation (Table 2). The average GC3s values for individual genomes varied from 65.84 to 89.35 in mycobacteriophage Barnyard and mycobacteriophage Cooper, respectively. In addition, there are marked intragenomic variations in Nc and GC3s values with standard deviation of >3.5 in both the indices. There seems to be a considerable heterogeneity in compositional bias and codon usage pattern within and among the genome of these phages. Of the 32 mycobacteriophages, the genome of Cooper is identified to have the lowest Nc and the highest GC3s values while Barnyard has the highest Nc and the lowest GC3s values indicating that highly GC rich genomes are more biased than poor GC rich genomes. 

In unicellular organisms, a strong correlation between gene expressivity and the extent of codon usage bias is reported for *Escherichia coli* and *Saccharomyces cerevisiae* and phages of *Staphycoccus aureus* and *mycobacteria* [13, 40, 44–48]. Our analysis reveals that the genome of mycobacteriophage Cooper is highly biased than other 31 mycobacteriophage genomes. Based on the comparison of the highly represented codons of cooper and the copy number of host specific tRNA, the data indicate that the putatively highly expressed genes of this phage have better translational efficiency comparatively and may be expressed rapidly by the host translation machinery.

Table 3 represents the base composition for the 32 completely sequenced mycobacteriophages. GC composition at the third codon position is always higher than the second and first + second codon positions observed in other GC-rich genomes [9–11]. It is also identified that there is major variation in GC3s content among the genomes studied with no major variation in GC1s and GC2s. This suggests that GC3s has a major role to play than GC1s and GC2s and is tightly associated with the codon usage bias of these genomes.

### 3.3. Synonymous Codon Usage in Different Mycobacteriophages

A plot of Nc versus GC3s (Nc plot) has been widely used to study the codon usage variation among genes in different organisms [19]. It was demonstrated that the comparison of the actual distribution of the genes, with the expected distribution under no selection, indicates that the codon usage bias of the genes has influences other than the compositional bias. In contrast, if GC3s were the only determinants of the codon usage variation among the genes, then the values of Nc would fall on the continuous curve between Nc and GC3s. 

**Figure 1:**
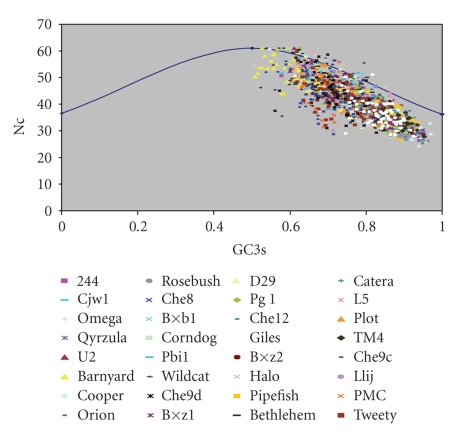
Nc plot of thirty two mycobacteriophages. The genes for individual phages are represented by different colors.

It is evident from Nc plot for the mycobacteriophages studied that most of the genes fall within a restricted cloud, at GC3s between 0.65 and 0.93, and Nc values 28 and 47 (Figure 1). Nc values for these genes lie just below the expected curve, indicating that these genes have additional codon usage bias apart from compositional bias. The rest of the genes have higher Nc values and lower GC3s values, mostly lying on and close to the expected curve. Consequently, the Nc values of these genes are substantially higher relative to expected values. However, strong influence of compositional constraints on codon usages bias in all the phages analyzed could be understood from the presence of significant negative correlation between GC3s and Nc (*r* = −0.969; *P* < .01).

### 3.4. Differential Base Usage in Third Codon Position

The correlation of the frequencies of four bases in the third position against Nc values of different genes of these 32 mycobacteriophages has been estimated (Table 4). As there is no information of gene expression level of mycobacteriophages so far, we have considered the highly biased genes having low Nc values as those highly expressed and vice versa. 

In all mycobacteriophages analyzed, except Che9d, the frequency of C at the third codon position increases with decreasing Nc values, whereas frequencies of T and A increase with Nc. However, the frequency of G is not influenced in the third codon position excluding for few phages such as Bxb1, Barnyard, Corndog, Plot, PBI1, and Bethelhem. Thus the influence of mutational bias of these phages is reflected in the choice of bases in the third position. However, this is expected since the optimal codons are, in general, chosen in accordance with the mutational bias of these phages. In other words, it is due to the translational selection that the mutational bias appears to be more prominent in the third codon position of highly expressed genes [18].

### 3.5. Effect of Translational Selection on the Synonymous Codon Usage in Mycobacteriophage Genomes

Some of the earlier reports showed that synonymous codon usage in the highly expressed genes of diverse array of organisms is influenced by cellular tRNA abundance [44, 49–52]. Kanaya et al. [52, 53] have reported that the cellular tRNA abundance in several organisms are directly proportional to their tRNA copy number.

Of the 32 mycobacteriophages analyzed, 10 phages (244 (2 tRNA genes), Bxz1 (26 tRNA genes), Omega (2 tRNA genes), Cjw1 (1 tRNA gene), Wildcat (21 tRNA genes), D29 (5 tRNA genes), L5 (3 tRNA genes), Che12 (3 tRNA genes), Catera (26 tRNA genes)) encode tRNA genes for few of the amino acids. Both Bxz1 and Catera are identified to encode large number of tRNA genes (26 tRNA genes) and Wildcat encoding for 21 tRNA genes. Of the 10 phage genomes encoding tRNA genes, excluding 244 and Omega, eight carry tRNA genes for the overrepresented codons in highly and lowly expressed genes. 

To see whether the synonymous codon usage of putatively highly expressed genes of these mycobacteriophage genomes is positively correlated with the host tRNA abundance, the number of over represented synonymous codons in such genes was determined by comparing with that of the putatively lowly expressed genes. It was found that among the 22 overrepresented synonymous codons in highly expressed genes, 21 codons are recognized by the *M.tuberculosis* specific tRNAs (data not shown). Based on the above analysis, the data indicate that the putatively highly expressed genes of these phages have translational efficiency.

### 3.6. Relationship between Codon Bias and Gene Length

**Figure 2:**
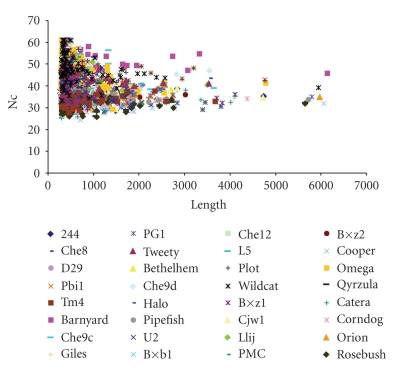
Plot of Nc versus Gene length for all mycobacteriophage genomes.

Selection for translational accuracy is predicted to have a positive correlation between codon bias and gene length [20]. Previously, the relationship between gene length and synonymous codon usage bias has been reported for *Drosophila melanogaster*, *Escherichia coli*, *Saccharomyces cerevisiae*, *Pseudomonas aeruginosa,* and *Yersinia pestis* [11, 15]. From the plot drawn with gene length against Nc (Figure 2), it is understood that shorter genes have a much wider variance in Nc values, and vice versa for longer genes. Lower Nc values in longer genes may be due to the direct effect of translation time on fitness or to the extra energy cost of proofreading associated with longer translating time. A significant correlation was identified in 11 phages revealing that gene length influences codon usage of these genomes (Table 5). Similar results were also reported for *S. pneumoniae*, *P. aeruginosa* and SARS coronavirus [11, 16]. Eyre-Walker [20] has reported that the selection for fidelity in protein translation is likely to be greater in longer genes because the cost of producing a protein is proportional to its length. Therefore selection of translational accuracy predicts a positive correlation between codon usage bias and gene length. And this selection may be stronger at constrained codons coding for evolutionarily conserved amino acids than the nonconserved amino acid. As the codon bias is lower in longer genes than shorter ones, further analysis in finding these constrained codons will help us in understanding whether it is the same in all genes irrespective of their length.

### 3.7. Correspondence Analysis Using RSCU Values

**Figure 3:**
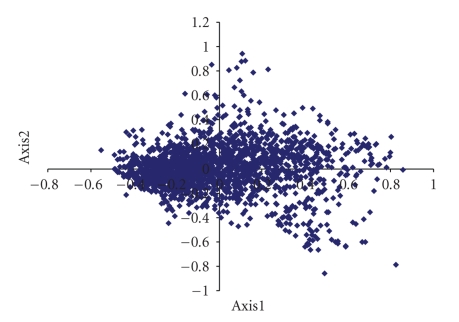
Correspondence analysis of Relative Synonymous Codon Usage values of mycobacteriophages (32 genomes).

In order to determine the factors that influence variations in codon usage among the genes of mycobacteriophage genomes, correspondence analysis was conducted on the RSCU values of its genes. Only the distributions of the genes along the first two major axes were shown, as these accounted for 13.63% and 6.89% of the total variation (Figure 3). 

**Figure 4:**
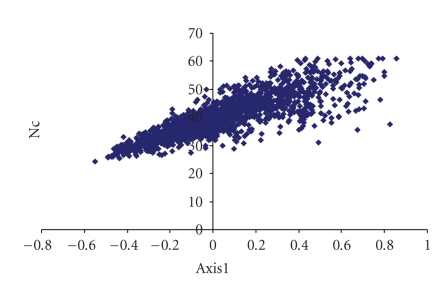
Scatter plot of mycobacteriophages and Nc values.

**Figure 5:**
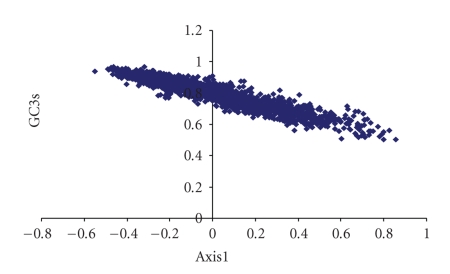
Scatter plot of mycobacteriophages and GC3s values.

The first major axis is negatively correlated with G3s (*r* = −.235, *P* < .01) and C3s (*r* = −.778, *P* < .01) but correlated positively with A3s (*r* = .687, *P* < .01) and T3s (*r* = .827, *P* < .01). Interestingly, high degree of positive correlation exists between position of genes along the first axis with Nc (*r* = .863, *P* < .01) (Figure 4) and high degree of negative correlation with GC3s (*r* = −.934, *P* < .01) (Figure 5). These findings suggest that highly biased genes, those with G- and C-ending codons, are clustered on the negative side, whereas the codons ending in A and T predominate on the positive side of the first major axis. 

Additionally, significant negative correlation is observed with Nc against GC3s and GC. Highly expressed genes tend to use “C” or “G” at the synonymous positions compared with lowly expressed genes. It is also studied that C-ending codons are preferred over G-ending codons in highly expressed genes. Preference of C-ending codons in the highly expressed genes might be related to the translational efficiency of the genes as it has been reported that RNY (R-purine, N- any nucleotide base, and Y-pyrimidine) codons are more advantageous for translation [54]. Thus, compositional mutation bias possibly plays an important role in shaping the genome of these phages. 

The genomes of Llij, PMC, Wildcat, TM4, Che8, Tweety, and Che9d phages showed no significant correlation between first major axis and GC3s. Whereas, phages such as 244, Bxb1, Bxz2, Che9c, Rosebush, Omega, Halo, Barnyard, Bxz1, Cjw1, Corndog, Orion, Plot, Qyrzula, and Giles show strong negative correlation with GC3s. The primary trend in codon usage variation in these phages can be attributed to the presence of putatively foreign genes acquired through horizontal gene transfer with unusually A + T rich codon usage. However, in phages such as D29, L5, PBI1, PG1, Cooper, Che12, Catera, Bethelhem, and U2, axis 1 coordinates are significantly positively correlated with GC3s values (Table 6). Moreover, when G3s and C3s are considered separately, the correlation coefficient exhibited by the positions of genes along the first axis with C3s is significantly larger than that with G3s (Table 6), indicating that the contribution of C3s to the interspecies variation in overall GC3s content is greater than that of G3s. 

Table 7 shows RSCU values for each codon for the two groups of genes. The asterisk represents the codons whose occurrences are significantly higher in the genes situated on the extreme left side of axis 1, compared to the genes present on the extreme right of the first major axis. It is important to note that out of 22 codons that are statistically overrepresented in genes located on the extreme left side of axis 1 there is 16 C-ending codons and 5-G ending codons. UGA is the most frequent stop codon among highly and lowly expressed genes. Similar pattern is also seen in *Mycobacterium tuberculosis* genome, where the highly expressed genes prefer codons ending with “C” and “G” [18].

## 4. Conclusion

Compositional bias and translational forces had been reported to play a major role in shaping the codon usage of 14 mycobacteriophages. Our observations corroborate with the earlier report with respect to all the 32 mycobacteriophages. Gene length has a minor role in the selection of codon usage of 11 out of 32 mycobacteriophage genes analyzed. High level of heterogeneity is seen within and among the mycobacteriophage genomes. Cooper is identified to be the highly biased genome with better translation efficiency comparing well with the host specific tRNA genes.

## Figures and Tables

**Table 1:** Relative synonymous codon usage for all the genes of 32 mycobacteriophages was calculated. AA and N are the amino acid and number of codons, respectively.

AA	Condon	N	RSCU	AA	Condon	N	RSCU
Phe	UUU	1795	(0.20)	Ser	UCU	2127	(0.40)
	UUC	15765	(1.80)		UCC	7032	(1.32)
Leu	UUA	195	0.03		UCA	1889	(0.35)
	UUG	5037	(0.66)		UCG	10888	(2.04)
Tyr	UAU	2447	(0.31)	Cys	UGU	874	(0.33)
	UAC	13288	(1.69)		UGC	4496	(1.67)
ter	UAA	393	(0.00)	Ter	UGA	1365	(0.00)
ter	UAG	388	(0.00)	Trp	UGG	11727	(1.00)
							
Leu	CUU	2805	(0.37)	Pro	CCU	3503	(0.42)
	CUC	13867	(1.82)		CCC	10146	(1.21)
	CUA	1400	(0.18)		CCA	2630	(0.31)
	CUG	22302	(2.93)		CCG	17369	(2.06)
His	CAU	2427	(0.40)	Arg	CGU	5190	(0.83)
	CAC	9611	(1.60)		CGC	16237	(2.60)
Gln	CAA	3076	(0.30)		CGA	3195	(0.51)
	CAG	17766	(1.70)		CGG	9987	(1.60)
							
Ile	AUU	3524	(0.40)	Thr	ACU	2990	(0.32)
	AUC	21992	(2.52)		ACC	20215	(2.20)
	AUA	636	(0.07)		ACA	2910	(0.32)
Met	AUG	11923	(1.00)		ACG	10698	(1.16)
Asn	AAU	2886	(0.29)	Ser	AGU	1693	(0.32)
	AAC	16738	(1.71)		AGC	8424	(1.58)
Lys	AAA	3185	(0.29)	Arg	AGA	692	(0.11)
	AAG	18997	(1.71)		AGG	2110	(0.34)
							
Val	GUU	4218	(0.40)	Ala	GCU	7738	(0.50)
	GUC	18361	(1.73)		GCC	26730	(1.72)
	GUA	1855	(0.17)		GCA	6591	(0.42)
	GUG	17981	(1.70)		GCG	21142	(1.36)
Asp	GAU	8082	(0.42)	Gly	GGU	10057	(0.78)
	GAC	30303	(1.58)		GGC	27920	(2.17)
Glu	GAA	8983	(0.50)		GGA	5241	(0.41)
	GAG	26657	(1.50)		GGG	8345	(0.65)

**Table 2:** Nc and GC3s values for 32 mycobacteriophages with standard deviation within brackets.

Phages	Nc	GC3s
244	38.98 (6.96)	80.65 (7.87)
Bxb1	40.17 (7.28)	80.05 (7.334)
Bxz2	37.93 (5.70)	82.72 (6.46)
Che9c	39.48 (6.96)	81.01 (7.842)
Rosebush	32.53 (4.04)	88.44 (4.27)
Omega	43.91 (6.54)	75.49 (7.01)
Halo	37.87 (5.24)	82.46 (5.59)
Barnyard	47.96 (7.24)	65.84 (8.87)
Bxz1	35.80 (5.64)	85.09 (6.48)
Cjw1	38.80 (6.40)	80.64 (7.83)
Corndog	37.87 (6.69)	82.65 (7.23)
Orion	37.79 (5.55)	82.85 (5.17)
Plot	44.28 (5.69)	73.35 (6.59)
Llij	43.52 (6.18)	72.23 (5.51)
Pipefish	35.12 (3.55)	87.35 (3.53)
PMC	42.62 (6.28)	71.95 (5.30)
Qyrzula	32.45 (3.93)	88.56 (4.37)
Wildcat	47.62 (6.63)	66.37 (6.29)
D29	37.83 (5.28)	82.58 (5.59)
L5	40.67 (5.70)	80.79 (5.86)
PBI1	44.07 (5.67)	73.71 (6.63)
PG1	37.59 (5.20)	82.7 (4.90)
Cooper	31.44 (4.65)	89.35 (5.90)
Che12	39.06 (4.83)	81.94 (5.75)
Catera	35.44 (5.66)	85.53 (6.24)
TM4	34.07 (3.60)	88.19 (4.64)
Che8	43.62 (6.33)	70.57 (5.37)
Tweety	42.76 (5.81)	72.07 (5.51)
U2	39.58 (7.03)	81.10 (7.26)
Bethlehem	40.22 (6.98)	80.35 (6.57)
Giles	36.48 (5.04)	83.38 (5.24)
Che9d	44.10 (7.13)	71.6 (5.8)

**Table 3:** GC % and base composition in the third codon position for 32 mycobacteriophage genomes.

Virus	G + C	GC1s	GC2s	GC (1st + 2nd)	GC3s	C3s	T3s	A3s	G3s
244	63.26	63.3	44.7	54	81	51.14	10.58	8.88	30.01
Bxb1	63.43	63.4	45.7	54.55	80.4	44.89	11.53	8.53	35.63
Bxz2	64.41	64	45.6	54.8	83	48.62	9.12	8.25	34.56
Che9C	65.66	66	48.9	57.45	81.4	47.27	10.17	8.9	34.16
Rosebush	69.16	68.3	49.8	59.05	88.5	51.21	6.26	5.36	37.73
Omega	61.48	62.4	45.1	53.75	76.1	45.43	13.88	10.78	30.52
Halo	63.79	63.4	44.5	53.95	82.7	47.4	9.57	8.06	35.5
Barnyard	58.05	60.9	45.5	53.2	67	38.55	18.39	15.97	27.68
Bxz1	65.04	64.1	44.9	54.5	85.3	51.84	9.61	5.39	33.72
Cjw1	63.52	63.7	45.1	54.4	81	51.39	10.2	9.27	29.76
Corndog	65.64	64.8	48.4	56.6	83	46.49	9.76	7.7	36.64
Orion	66.9	66.6	50.2	58.4	83.17	50.46	10.19	7.04	32.85
Plot	60.22	61.6	44.3	52.95	74.1	42.4	15.73	11.05	31.34
Llij	61.66	62.4	48.9	55.65	73.1	39.73	16.12	11.79	32.87
Pipefish	67.64	65.4	49.3	57.35	87.5	49.1	7.19	5.51	38.72
PMC	61.46	62.8	48.2	55.5	72.8	39.67	15.81	12.38	32.66
Qyrzula	69.14	68.1	49.8	58.95	88.6	51.07	6.08	5.41	38
Wildcat	57.03	59.8	42.8	51.3	67.7	35.37	22.27	11.57	31.45
D29	63.78	63.4	44.4	53.9	82.9	47.52	9.51	8	35.49
L5	62.49	62.1	43.5	52.8	81.2	47.45	10.01	9.3	33.79
PBI1	60.28	61.4	44.3	52.85	74.5	42.71	15.41	10.99	31.37
PG1	66.83	66.5	50.2	58.35	83.12	50.46	10.24	7.07	32.79
Cooper	69.26	67.6	50	58.8	89.47	52.65	6.32	4.38	37.19
Che12	63.26	62.5	44.4	53.45	82.25	48.61	9.86	8.3	33.79
Catera	65.17	64.1	44.9	54.5	85.8	52.29	9.44	5.1	33.71
Che8	61.41	63.71	48.6	56.16	70.57	37.08	16.05	12.52	34.32
TM4	68.7	68.3	48.9	58.6	88.19	47.26	6.66	5.08	40.99
Che9d	61.4	63.02	48.15	55.59	71.6	37.07	15.54	11.94	35.41
Bethlehem	63.29	63.37	45.22	54.30	80.35	42.56	11.32	7.93	38.15
Giles	67.89	66.55	52.72	59.64	83.38	48.11	6.26	10.06	35.55
Tweety	61.83	63.12	48.89	56.01	72.07	38.77	14.85	12.15	34.2
U2	63.87	63.61	45.92	54.77	81.1	43.53	10.68	7.83	37.94

**Table 4:** Correlation coefficient of Nc with C3s, T3s, A3s, and G3s base composition.

	NC			
Phage name	C3s	T3s	A3s	G3s
244	−.814**	.816**	.655**	−0.126^NS^
Bxb1	−.509**	.727**	.867**	−.532**
Bxz2	−.631**	.636**	.829**	−.315*
Che9C	−.705**	.829**	.831**	−0.19^NS^
Rosebush	−.599**	.688**	.609**	−0.063^NS^
Omega	−.714**	.635**	.618**	−0.141^NS^
Halo	−.564**	.394**	.690**	−0.129^NS^
Barnyard	−.641**	.694**	.796**	−.621**
Bxz1	−.742**	.725**	.777**	−0.123^NS^
Cjw1	−.808**	.774**	.629**	−0.104^NS^
Corndog	−.484**	.788**	.621**	−.320**
Orion	−.817**	.726**	.673**	0.147^NS^
Plot	−.666**	.709**	.717**	−.401**
Llij	−.278*	.348**	0.212^NS^	−0.041^NS^
Pipefish	−.372**	.527**	.567**	−0.085^NS^
PMC	−.442**	.409**	−0.047	0.247^NS^
Qyrzula	−.657**	.743**	.665**	−0.048^NS^
Wildcat	−0.216^NS^	0.069^NS^	.487**	−.274*
D29	−.564**	.391**	.690**	−0.13^NS^
L5	−.642**	.668**	.735**	−0.182^NS^
PBI1	−.662**	.718**	.702**	−.387**
PG1	−.815**	.735**	.616**	0.214^NS^
Cooper	−.672**	.806**	.844**	−0.165^NS^
Che12	−.711**	.708**	.628**	−0.115^NS^
Catera	−.716**	.768**	.702**	−0.125^NS^
TM4	−.575**	.635**	.601**	−0.021^NS^
Bethlehem	−.583**	.700**	.758**	−.468**
Che8	−.404**	.410**	0.036^NS^	0.109^NS^
Tweety	−.359**	.353**	0.106^NS^	0.088^NS^
Giles	−.535**	.502**	.406**	0.158^NS^
U2	−.514**	.783**	.880**	−.544**
Che9d	−0.102^NS^	0.085	.401**	−0.194^NS^

Notable significant relationships are marked by **P < .01 or *P < .05, ^NS^Nonsignificant.

**Table 5:** Correlation coefficient of gene length with Nc and GC3s values.

	Length	
Phage name	Nc	GC3s
Bxb1	−.440**	.445**
Bxz2	−.328*	.414**
Omega	−.223*	.243**
Bxz1	−.241**	.205*
Cjw1	−.227*	0.213^NS^
Corndog	−.331**	.336**
Llij	−.342**	.332**
Wildcat	−.280*	0.026^NS^
Catera	−.200*	.168*
U2	−.475**	.427**
Bethlehem	−.472**	.445**

Notable significant relationships are marked by **P < .01 or *P < .05, ^NS^Nonsignificant.

**Table 6:** Correlation of Axis1 with other codon usage indices.

	Axis 1						
Virus	GC3s	A3s	T3s	G3s	C3s	Gravy	Aromaticity
244	−.930**	.729**	.829**	−.230*	−.798**	−0.206^NS^	0.178^NS^
Bxb1	−.891**	.871**	.689**	−.417**	−.603**	−0.222^NS^	0.078^NS^
Bxz2	−.904**	.865**	.676**	−.503**	−.502**	−.323*	−0.16^NS^
Che9C	−.921**	.854**	.848**	−0.201^NS^	−.717**	−0.002^NS^	−0.053^NS^
Rosebush	−.689**	.323**	.743**	0.108^NS^	−.638**	0.151^NS^	0.028^NS^
Omega	−.897**	.650**	.771**	−.262**	−.736**	0.004^NS^	−0.055^NS^
Halo	−.746**	.720**	.498**	−0.125^NS^	−.641**	−0.091^NS^	0.013^NS^
Barnyard	−.933**	.843**	.792**	−.647**	−.730**	0.02^NS^	0.023^NS^
Bxz1	−.901**	.859**	.729**	−.221**	−.718**	0.005^NS^	−0.048^NS^
Cjw1	−.929**	.796**	.742**	−.362**	−.712**	−.259*	0.153^NS^
Corndog	−.904**	.647**	.838**	−0.175^NS^	−.652**	−0.184^NS^	−0.002^NS^
Orion	−.763**	.641**	.644**	0.099^NS^	−.729**	−.362**	−0.169^NS^
Plot	−.826**	.820**	.594**	−.427**	−.631**	0.172^NS^	0.229^NS^
Llij	0.15^NS^	.763**	−.846**	−.912**	.896**	−0.186^NS^	−0.166^NS^
Pipefish	.370**	−0.205^NS^	−.348**	−0.036^NS^	.263*	−0.105^NS^	0.185^NS^
PMC	0.097^NS^	.853**	−.919**	−.944**	.908**	−0.229^NS^	−0.078^NS^
Qyrzula	−.641**	.323**	.731**	0.09^NS^	−.588**	0.061^NS^	−0.076^NS^
Wildcat	0.14^NS^	−.788**	.556**	.411**	−0.208^NS^	0.017^NS^	−0.036^NS^
D29	.740**	−.721**	−.479**	0.126^NS^	.634**	0.241^NS^	−0.041^NS^
L5	.862**	−.775**	−.670**	.384**	.485**	.296*	−0.071^NS^
PBI1	.845**	−.806**	−.664**	.365**	.705**	−0.131^NS^	−.269*
PG1	.740**	−0.524^NS^	−.678**	−0.219^NS^	.753**	.277**	0.118^NS^
Cooper	.782**	−.782**	−.609**	0.153^NS^	.563**	0.2^NS^	−0.001^NS^
Che12	.803**	−.752**	−.596**	0.201^NS^	.650**	0.214^NS^	−0.249^NS^
Catera	.914**	−.840**	−.766**	.233**	.702**	0.041^NS^	0.002^NS^
TM4	0.085^NS^	*‒*.313*	0.258^NS^	0.182^NS^	−0.094^NS^	0.003^NS^	0.008^NS^
Bethelhem	.882**	−.881**	−.625**	.505**	.602**	0.229^NS^	.294*
Che8	0.124^NS^	.816**	−.883**	−.917**	.899**	−0.242^NS^	−0.189^NS^
Tweety	−0.113^NS^	−.797**	.891**	.934**	−.902**	0.231^NS^	0.098^NS^
Giles	−.352**	.730**	−0.447^NS^	−.887**	.691**	−.444**	−0.102^NS^
U2	.922**	−.918**	−.737**	.522**	.537**	0.066^NS^	0.01^NS^
Che9d	0.16^NS^	.778**	−.831**	−.857**	.862**	−0.027^NS^	−0.231^NS^

Notable significant relationships are marked by **P < .01 or *P < .05, ^NS^Nonsignificant.

**Table 7:** Relative Synonymous Codon Usage for the highly and lowly expressed genes.

AA	Codon	RSCU^a^	N^a^	RSCU^b^	N^b^	AA	Codon	RSCU^a^	N^a^	RSCU^b^	N^b^
Phe	UUU	0.08	(34)	0.57	(185)	Ser	UCU	0.11	(27)	0.94	(178)
	UUC*	1.92	(839)	1.43	(466)		UCC*	1.20	(284)	0.88	(166)
											
Leu	UUA	0.00	(0)	0.16	(41)		UCA	0.10	(23)	0.84	(160)
	UUG	0.09	(33)	1.55	(390)		UCG	1.76	(417)	1.67	(317)
	CUU	0.07	(26)	0.93	(235)	Pro	CCU	0.16	(60)	0.85	(249)
	CUC*	1.38	(518)	1.16	(292)		CCC*	1.49	(545)	0.88	(257)
	CUA	0.01	(5)	0.49	(123)		CCA	0.12	(43)	0.71	(209)
	CUG*	4.45	(1676)	1.70	(429)		CCG*	2.23	(813)	1.56	(459)
											
Ile	AUU	0.19	(78)	1.01	(288)	Thr	ACU	0.09	(47)	0.78	(217)
	AUC*	2.81	(1181)	1.74	(495)		ACC*	3.11	(1573)	1.20	(333)
	AUA	0.00	(1)	0.25	(72)		ACA	0.07	(33)	0.70	(193)
Met	AUG	1.00	(648)	1.00	(427)		ACG	0.73	(367)	1.32	(364)
											
Val	GUU	0.15	(88)	0.92	(332)	Ala	GCU	0.22	(183)	0.91	(428)
	GUC*	1.97	(1131)	1.04	(373)		GCC*	2.50	(2078)	1.03	(483)
	GUA	0.08	(48)	0.34	(123)		GCA	0.22	(181)	0.76	(355)
	GUG	1.79	(1025)	1.69	(608)		GCG	1.06	(882)	1.30	(611)
											
Tyr	UAU	0.10	(41)	0.69	(185)	Cys	UGU	0.06	(7)	0.83	(123)
	UAC*	1.90	(767)	1.31	(350)		UGC*	1.94	(212)	1.17	(175)
											
TER	UAA	0.56	(20)	0.79	(28)	TER	UGA	1.77	(63)	1.63	(58)
	UAG	0.67	(24)	0.59	(21)	Trp	UGG	1.00	(531)	1.00	(468)
											
His	CAU	0.12	(36)	0.89	(236)	Arg	CGU	0.52	(164)	1.37	(364)
	CAC*	1.88	(570)	1.11	(296)		CGC*	3.79	(1189)	1.50	(399)
											
Gln	CAA	0.06	(29)	0.63	(221)		CGA	0.18	(55)	0.85	(227)
	CAG*	1.94	(1021)	1.37	(480)		CGG	1.41	(442)	1.23	(328)
											
Asn	AAU	1.10	(50)	0.75	(265)	Ser	AGU	0.11	(27)	0.73	(139)
	AAC*	1.90	(926)	1.25	(446)		AGC*	2.71	(642)	0.94	(178)
											
Lys	AAA	0.06	(33)	0.60	(229)	Arg	AGA	0.00	(1)	0.41	(109)
	AAG*	1.94	(1046)	1.40	(539)		AGG	0.11	(33)	0.64	(170)
											
Asp	GAU	0.15	(151)	0.89	(564)	Gly	GGU	0.54	(305)	1.04	(414)
	GAC*	1.85	(1868)	1.11	(706)		GGC*	2.90	(1646)	1.19	(471)
Glu	GAA	0.29	(270)	0.84	(513)		GGA	0.14	(81)	0.91	(363)
	GAG*	1.71	(1609)	1.16	(704)		GGG	0.41	(235)	0.86	(341)

*Codons whose occurrences are significantly higher (*P* < .01) in the extreme left side of axis 1 than the genes present on the extreme right of the first major axis. Each group contains 10% of sequences at either extreme of the major axis generated by correspondence analysis. AA: amino acid; N: number of codon; ^a^genes on extreme left of axis 1; ^b^genes on extreme right of axis 1.
